# Tripeptide tyroserleutide plus doxorubicin: therapeutic synergy and side effect attenuation

**DOI:** 10.1186/1471-2407-8-342

**Published:** 2008-11-25

**Authors:** Zhi-feng Zhu, Li-juan Chen, Rong Lu, Jing Jia, Yu Liang, Qiong Xu, Chun-lei Zhou, Li Wang, Song Wang, Zhi Yao

**Affiliations:** 1 Department of Immunology, Tianjin Medical University, Tianjin 300070, PR China; 2 Shenzhen Kangzhe Pharmaceutical Co. Ltd., Shenzhen 518057, PR China; 3 Department of Histology and Embryology, Tianjin Medical University, Tianjin 300070, PR China; 4 Tianjin Key Laboratory of Cellular and Molecular Immunology, Tianjin Medical University, Tianjin 300070, PR China

## Abstract

**Background:**

Tripeptide tyroserleutide (YSL) is a novel small molecule anti-tumor polypeptide that has been shown to inhibit the growth of human liver cancer cells. In this study, we investigated the effects of YSL plus doxorubicin on the growth of human hepatocellular carcinoma BEL-7402 cells that had been transplanted into nude mice.

**Methods:**

Nude mice bearing human hepatocellular carcinoma BEL-7402 tumors were treated with successive intraperitoneal injections of saline; low-, mid-, or high-dose doxorubicin; or low-, mid-, or high-dose doxorubicin plus YSL. Effects on the weight and volume of the tumors were evaluated.

**Results:**

Co-administration of YSL and high-dose doxorubicin (6 mg/kg every other day) prolonged the survival time of tumor-bearing mice as compared to high-dose doxorubicin alone. As well, the anti-tumor effects of mid- and low-dose doxorubicin (2 and 0.7 mg/kg every other day, respectively) were enhanced when supplemented with YSL; the tumor growth inhibition rates for YSL plus doxorubicin were greater than the inhibition rates for the same dosages of doxorubicin alone. The combination of YSL and doxorubicin decreased chemotherapy-associated weight loss, leukocyte depression, and heart, liver, and kidney damage as compared to doxorubicin alone.

**Conclusion:**

The combination of YSL plus doxorubicin enhances the anti-tumor effect and reduces the side effects associated with doxorubicin chemotherapy.

## Background

According to the World Health Organization, 7.6 million people succumbed to cancer in 2005, accounting for 13% of all deaths. However, in China, the cancer death rate was 20.2%, and it is expected to reach 23.6% by 2023. Liver cancer is the third most common cause of death in China [1].

Anthracene-nucleus antibiotics are anti-tumor drugs that have been heavily researched and developed for the past twenty years. Among those, doxorubicin is the most commonly used and the most important; however, its use has been increasingly limited due to severe associated side effects, such as myocardial toxicity and bone marrow depression [2]. Because the dosage of doxorubicin can be decreased when doxorubicin is given combination with other anti-tumor drugs, which can also elevate the therapeutic effect and relieve side effects of doxorubicin, combination therapy has become a common strategy for doxorubicin-based chemotherapy.

Tripeptide tyroserleutide (YSL) is a small molecule polypeptide that consists of three natural amino acids: L-tyrosine, L-serine, and L-leucine. In our laboratory, we have observed anti-tumor activity for YSL both *in vitro *and *in vivo *using the MTT method, the mouse model of ascites fluid-type hepatocarcinoma H22, and the nude mouse model of human hepatocellular carcinoma [3,4]. In this study, we established a nude mouse model of human hepatocellular carcinoma in order to investigate the effect of YSL on tumor growth when given in combination with doxorubicin.

## Methods

### Cell culture

Human hepatocellular carcinoma BEL-7402 cells were purchased from Institute of Cell Biology, Shanghai, Academia Sinica. The cells were cultured in RPMI 1640 supplemented with 10% fetal bovine serum in a humidified atmosphere of 5% CO_2 _at 37°C. During cultivation, BEL-7402 cells remained free of mycoplasma or bacterial contamination.

### Animals

Healthy female BALB/c (nu/nu) mice (4–5 weeks old, 18–22 g) were obtained from Chinese Academy of Medical Sciences (Beijing, China). The animals were housed at our university under specific pathogen-free conditions according to the guidelines of the Chinese Association for Laboratory Animal Care, using a laminar airflow rack. Animals had continuous access to sterilized food pellets and autoclaved water, and a 12-hr light/dark cycle. The temperature was maintained at 22–26°C and 50–70% relative humidity. All studies were performed according to the regulations of Tianjin Medical University experimental animal center and obtained the ethical approval from Tianjin Council for the Care of Animal in Medical Research.

### Nude mouse model of human hepatocellular carcinoma [5]

To establish the human hepatocellular carcinoma model, BEL-7402 cells in exponential phase were grown to a density of 1 × 10^8^/ml. 0.1 ml of the cell suspension was injected subcutaneously into the right back of every nude mouse. Mice with tumors of uniform volume were selected and randomly divided into different treatment groups when the tumor volume reached 100 mm^3^.

### YSL plus high-dose doxorubicin combination therapy

YSL was obtained from Shenzhen Kangzhe Pharmaceutical, China. Doxorubicin (Adriamycin, ADM) was obtained from Zhejiang Haizheng Pharmaceutical Co., Ltd.

Nude mice bearing tumors were randomly divided into high-dose doxorubicin (6 mg/kg every other day), YSL (320 μg/kg/d), YSL (320 μg/kg/d) plus high-dose doxorubicin (6 mg/kg every other day), and saline (0.2 ml/d) groups; each group had twenty mice. All medicines were dissolved in 0.2 ml saline. Doxorubicin was administered intraperitoneally (IP) every other day, YSL and saline (control) were administered every day. For animals receiving combination therapy, the two medications were administered at a 4-hr interval. Mice received successive IP administrations for 30 days or until the death of mouse. Beginning on the day after the first administration, we observed activity, stool color and stool patterns of the mice in all treatment groups. We recorded the survival status of mice daily and calculated the rate of life extension according to the following formula: the rate of life extension = (average survival days of experiment group-average survival days of control group)/average survival days of control group × 100%.

### YSL plus mid-dose doxorubicin combination therapy

Nude mice bearing tumors were randomly divided into mid-dose doxorubicin (2 mg/kg every other day), YSL (320 μg/kg/d), YSL (320 μg/kg/d) plus mid-dose doxorubicin (2 mg/kg every other day), and saline (0.2 ml/d) groups; each group had twelve mice. Mice received IP administrations for 30 successive days. The weights of mice were measured every three days until the experiment was terminated.

On the day after treatment termination, blood samples were obtained from the angular vein and anticoagulated with heparin. We measured hemoglobin levels, blood platelets count, and leukocyte counts. Hearts, livers, and kidneys were fixed immediately in 10% formalin. Paraffin-embedded sections of each tissue sample were stained with hematoxylin and eosin (HE). The weight and three perpendicular diameters of every tumor were recorded. Tumor volume was calculated by the following formula: V = (1/6) π ABC, where V is the tumor volume, and A, B, and C are the three tumor diameters. The rate of tumor inhibition = (average tumor weight of control group – average tumor weight of experiment group)/average tumor weight of control group × 100%.

### YSL plus low-dose doxorubicin

Nude mice bearing tumors were randomly divided into low-dose doxorubicin (0.7 mg/kg every other day), YSL (320 μg/kg/d), YSL (320 μg/kg/d) plus low-dose doxorubicin (0.7 mg/kg every other day), and saline (0.2 ml/d) groups; each group had twelve mice. Treatments were administered IP for 60 successive days. Mouse characteristics including body weights, blood counts, and tumor volumes and weights were observed and recorded as described in the study of mid-dose doxorubicin.

### Statistical Analysis

Data are expressed as mean ± standard deviation (SD). Statistical significances of survival time differences were analyzed according to the Kaplan-Meier method using SPSS software. The statistical significance of other differences was tested using one-way analysis of variance (ANOVA) followed by the Student-Newman-Keuls test. Statistical significance was set at P < 0.05.

## Results

### YSL plus high-dose doxorubicin prolongs the survival time of nude mice bearing tumors compared with doxorubicin alone

After about 10 days of administration, the mice that received high-dose doxorubicin alone showed weight loss and decreased activity level. Similar changes were observed in the YSL plus doxorubicin group; however, in general, the condition of the combination group was better than that of the doxorubicin group. No obvious side effects were observed in YSL group.

**Figure 1:**
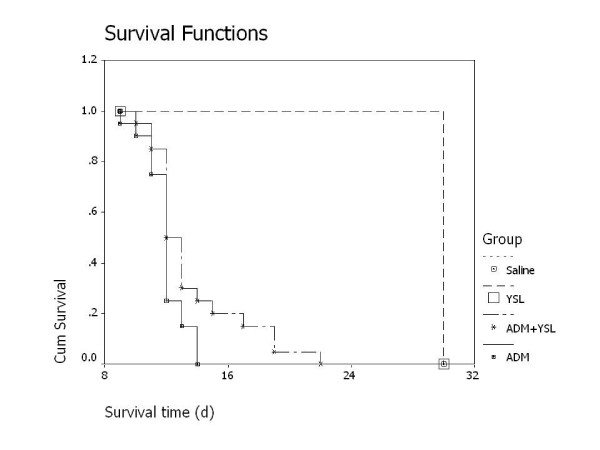
**Survival times of nude mice bearing human hepatocellular carcinoma BEL-7402 tumors treated with either high-dose doxorubicin or YSL plus high-dose doxorubicin**. BEL-7402 cell suspension (1 × 10^7^/each mouse) was subcutaneously inoculated into the right back of every nude mouse. When tumor volume research 100 mm^3^, they were randomly divided into different groups (n = 20). The mice were administrated i.p. for 30 days or until the death of mouse. Statistical significances of survival time were tested by Kaplan-Meier method using SPSS, and difference between the group of high dosage of ADM and the group of YSL combination group was tested by Log Rank, statistical significance was set at *P *< 0.05. ADM, doxorubicin or adriamycin; YSL, tripeptide tyroserleutide.

All the animals in YSL group and saline group were still alive 30 days later (termination of the experiments). The mean survival time of mice that received YSL plus high-dose doxorubicin was longer than that of mice that received high-dose doxorubicin alone. This difference was significant, and the extension rate of survival time was 16.67% (P < 0.05; Figure 1).

### Co-administration of YSL diminishes the side effects associated with mid-dose doxorubicin

**Figure 2:**
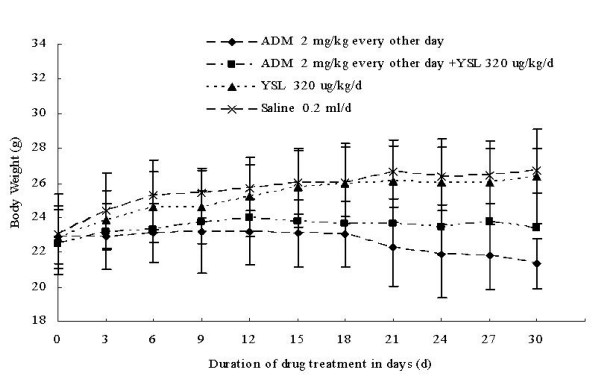
**The effects of YSL plus mid-dose doxorubicin on the weight of nude mice bearing human hepatocellular carcinoma BEL-7402 tumors**. BEL-7402 cell (1 × 10^7^/every mouse) was subcutaneously inoculated into the right back of every nude mouse. When the tumors had reached an average volume of 100 mm^3^, the tumor-bearing nude mice were randomly divided into different groups (n = 12). Drugs delivered by i.p. injection for 30 days successively. We weighted every group mice by electronic balance every 3 day and recorded the weight. Data points represent mean body weight ± SD. ADM, doxorubicin or adriamycin; YSL, tripeptide tyroserleutide.

After about 12 days, the mice treated with mid-dose doxorubicin alone showed decreased body temperature and lackluster hair. Similar changes were observed in the YSL combination group, but in general, their condition was better than that of the doxorubicin group. The body temperature of the mice that received YSL and doxorubicin was lower than that of the mice that received doxorubicin alone. Mice in YSL group and saline group were in good physical condition and showed weight gain. There were no significant difference in weight gain between YSL group and saline group. The weight of the mice in YSL plus mid-dose doxorubicin and mid-dose doxorubicin alone was lower after drug administration than before administration. However, the mean weight of the YSL combination group was higher than the mean weight of the doxorubicin group, and the mean weight loss for YSL combination group was lower than that of the doxorubicin group. As well, the weight loss in YSL combination group manifested later than in the doxorubicin group (Figure 2).

**Figure 3:**
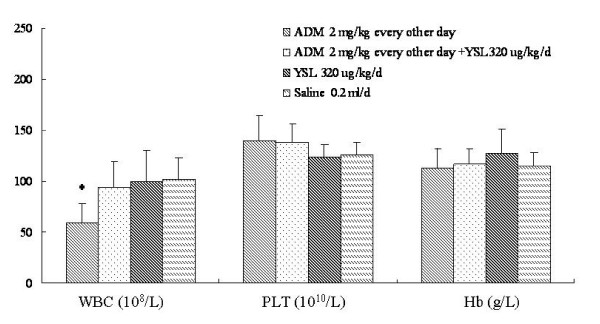
**The effects of YSL plus mid-dose doxorubicin on the hematological indices of nude mice bearing human hepatocellular carcinoma BEL-7402 tumors**. BEL-7402 cell (1 × 10^7^/every mouse) was subcutaneously inoculated into the right back of every nude mouse. When the tumors had reached an average volume of 100 mm^3^, the tumor-bearing nude mice were randomly divided into different groups (n = 12). Drugs delivered by i.p. injection for 30 days successively. At the next day of the last administration, blood was obtained from angular vein of nude mice, then blood was anticoagulated by heparin, we detected hematoglobin level and counted PLT and WBC. All data were analyzed by one-way analysis of variance using SPSS. The difference between two groups was analyzed by the Student-Newman-Keuls test. Standard deviation was represented by error line. *P < 0.05 as compared to the saline group. ADM, doxorubicin or adriamycin; YSL, tripeptide tyroserleutide. WBC, white blood cells; PLT, platelets; Hb, hemoglobin.

There were no significant difference in hemoglobin levels, blood platelets count and between YSL group and saline group. Leukocyte counts were markedly lower in the doxorubicin group than in the YSL combination group and YSL alone group, and were significantly lower as compared to the saline group (P < 0.05). Blood platelet counts were higher in doxorubicin group than in both the YSL combination and saline groups, but there was no statistical significance versus the saline group (P > 0.05). Hemoglobin levels in both the doxorubicin and YSL combination groups were higher than in the saline group, but these differences were not statistically significant (Figure 3).

**Figure 4:**
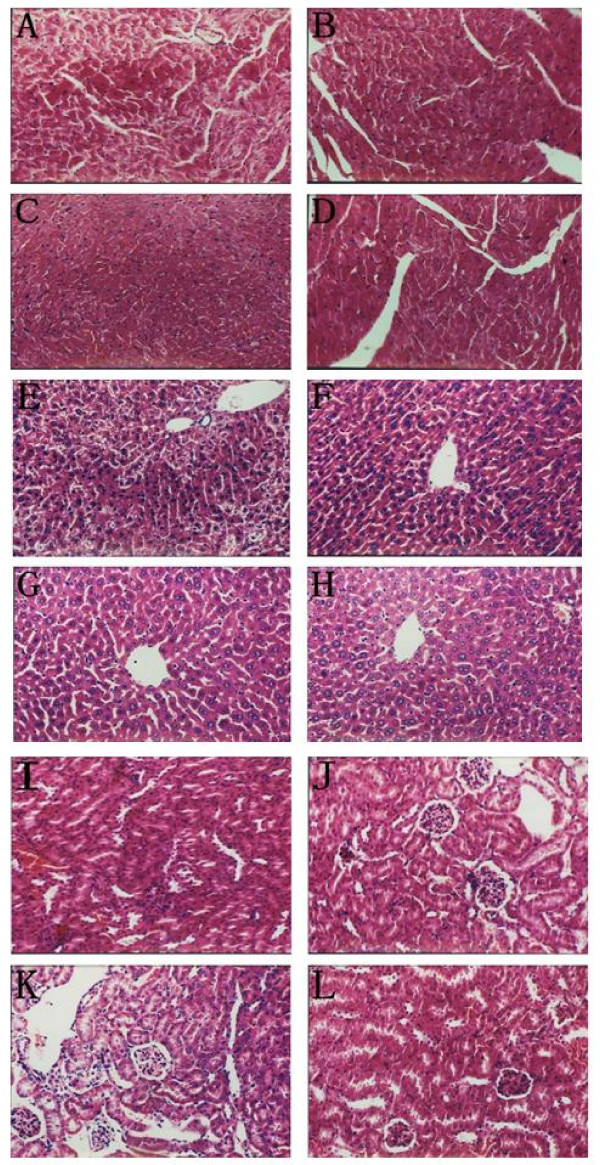
**The effects of YSL plus mid-dose doxorubicin on the internal organs of nude mice bearing human hepatocellular carcinoma BEL-7402 tumors**. BEL-7402 cell (1 × 10^7^/every mouse) was subcutaneously inoculated into the right back of every nude mouse. When the tumors had reached an average volume of 100 mm^3^, the tumor-bearing nude mice were randomly divided into different groups (n = 12). The mice were administrated i.p. 30 days. At the next day of the last administration, nude mice were sacrificed, and then put the heart, liver and kidney tissue into 10% formalin to fix and paraffin-embedded sections of each tissue sample were stained routinely with hematoxylin and eosin (HE). Figures 4A, 4B, 4C and 4D are heart samples. Figures 4E, 4F, 4G, and 4H are liver samples. Figures 4I, 4J, 4K and 4L are kidney samples. Figure 4A (Mid-dose doxorubicin): Myocardial cells are pultaceous and characterized by enhanced acidophilia. Figure 4B (YSL plus mid-dose doxorubicin): The transverse striation of myocardial cells is clear and with good repair. Figure 4C (YSL group) and Figure 4D (Saline group): There are no obvious abnormalities. Figure 4E (Mid-dose doxorubicin): There was enhanced acidophilia and interstitial edema. Figure 4F (YSL plus mid-dose doxorubicin): The structure of hepatic lobules is clear. Figure 4G (YSL group) and Figure 4H (Saline group): There are no obvious abnormalities. Figure 4I (Mid-dose doxorubicin): There are congestion of renal glomeruli and narrowed Bowman's space. Figure 4J (YSL plus mid-dose doxorubicin): Nephric tubules exhibit swelling. Figure 4K (YSL group) and Figure 4L (Saline group): There are no obvious abnormalities.

Including YSL in the treatment regimen decreased the toxic effects of mid-dose doxorubicin on the heart, liver, and kidney of nude mice bearing tumors as shown by HE staining (Figure 4). In the mid-dose doxorubicin group, there was puffing of cardiac muscle cells, enhanced acidophilia, and augmented interspaces. The presence of these pathological changes was softened in the YSL combination group, in which the structure of heart muscle was comparatively complete. The structure of hepatic lobules was not clear in the mid-dose doxorubicin group, and there was enhanced acidophilia and interstitial edema. However, in the YSL combination group the structure of the hepatic lobules was clear. The structure of the kidney cortical labyrinth and medullary ray was acceptable in the mid-dose doxorubicin group, but the cell proliferation of the glomeruli decreased, there was party acinus renalis blood capillary glomus congestion, narrowed Bowman's space, and interstitial congestion. These effects were muted slightly in the YSL combination group. There are no obvious pathological changes in YSL alone and saline group.

**Figure 5:**
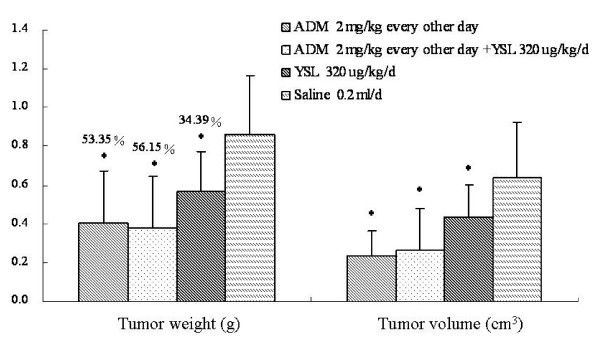
**The effects of YSL plus mid-dose doxorubicin on tumor growth in nude mice bearing human hepatocellular carcinoma BEL-7402 tumors**. BEL-7402 cell (1 × 10^7^/every mouse) was subcutaneously inoculated into the right back of every nude mouse. When the tumors had reached an average volume of 100 mm^3^, the tumor-bearing nude mice were randomly divided into different groups (n = 12). The mice were administrated i.p. 30 days. At the next day of the last administration, tumor tissue were stripped and three diameter which were vertical each other were measured. The tumor volume was calculated by V (cm^3^) = (1/6) π ABC, and A, B, C was three diameter of tumor. Then we weigh the tumor and calculated the tumor inhibition rate by tumor inhibition rate= (average weight of control group-average weight of experimental group)/average weight of control group × 100%. All data were analyzed by one-way analysis of variance using SPSS. The difference between two groups was analyzed by the Student-Newman-Keuls test. Standard deviation was represented by error line. *P < 0.05 as compared to the saline group. ADM, doxorubicin or adriamycin; YSL, tripeptide tyroserleutide.

YSL alone and mid-dose doxorubicin could inhibit tumor growth in nude mice bearing human hepatocellular carcinoma BEL-7402 tumors. The tumor inhibition rate was 34.39% and 53.35%, respectively, which were statistically significant as compared with the saline group (P < 0.05). The tumor inhibition rate for combination therapy was 56.15%, which was statistically significant as compared to the saline group (P < 0.05). However, there was no significance difference between the inhibition rates of the YSL combination group and the doxorubicin group (P > 0.05; Figure 5).

### The anti-tumor effects of YSL plus low-dose doxorubicin

**Figure 6:**
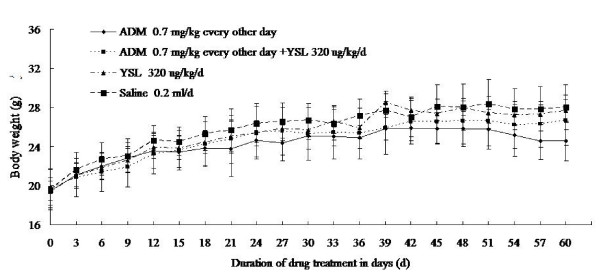
**The effects of YSL plus low-dose doxorubicin on the body weight of nude mice bearing human hepatocellular carcinoma BEL-7402 tumors**. BEL-7402 cell (1 × 10^7^/every mouse) was subcutaneously inoculated into the right back of every nude mouse. When the tumors had reached an average volume of 100 mm^3^, the tumor-bearing nude mice were randomly divided into different groups (n = 12). The mice were administrated i.p. 60 days. The body weights were weighing by electronic balance every three day. Standard deviation was represented by error line. ADM, doxorubicin or adriamycin; YSL, tripeptide tyroserleutide.

Mice in four groups, including YSL group, YSL plus low-dose doxorubicin group, low-dose doxorubicin group and saline group, were in good physical condition and showed weight gain. Mice treated with YSL group or low-dose doxorubicin (0.7 mg/kg every other day) or YSL plus low-dose doxorubicin tolerated therapy well, there were no overt side effects in either group. The mean body weight of nude mice in the YSL combination group was higher than that in doxorubicin group, but this difference was not significant (P > 0.05; Figure 6).

**Figure 7:**
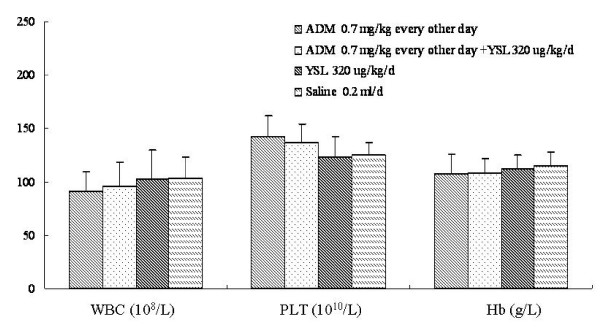
**The effects of YSL combination with low dosage of doxorubicin on hematological indices of nude mice bearing human hepatocellular carcinoma BEL-7402 tumors**. BEL-7402 cell (1 × 10^7^/every mouse) was subcutaneously inoculated into the right back of every nude mouse. When the tumors had reached an average volume of 100 mm^3^, the tumor-bearing nude mice were randomly divided into different groups (n = 12). Drugs delivered by i.p. injection for 60 days successively. At the next day of the last administration, blood was obtained from angular vein of nude mice, then blood was anticoagulated by heparin, we detected hematoglobin level and counted PLT and WBC. All data were analyzed by one-way analysis of variance using SPSS. The difference between two groups was analyzed by the Student-Newman-Keuls test. Standard deviation was represented by error line. Statistical significance was set at *P *< 0.05. ADM, doxorubicin or adriamycin; YSL, tripeptide tyroserleutide. WBC, white blood cells; PLT, platelets; Hb, hemoglobin.

There were no obvious differences in white blood cell counts, platelet counts, or hemoglobin levels among the three treatment groups, and there were no significance differences as compared to the saline group (P > 0.05; Figure 7).

**Figure 8:**
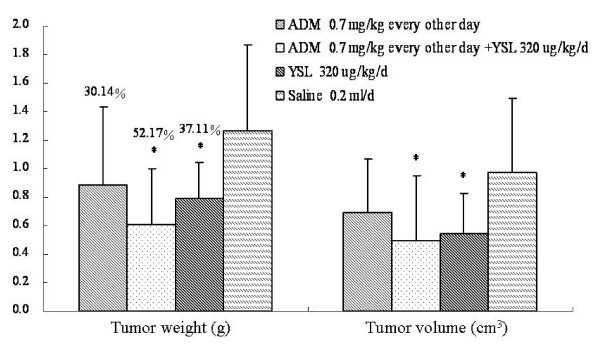
**The effects of YSL plus low-dose doxorubicin on tumor growth in nude mice bearing human hepatocellular carcinoma BEL-7402 tumors**. BEL-7402 cell (1 × 10^7^/every mouse) was subcutaneously inoculated into the right back of every nude mouse. When the tumors had reached an average volume of 100 mm^3^, the tumor-bearing nude mice were randomly divided into different groups (n = 12). The mice were administrated i.p. 60 days. At the next day of the last administration, tumor tissue were stripped and three diameter which were vertical each other were measured. The tumor volume was calculated by V (cm^3^) = (1/6)π ABC, and A, B, C was three diameter of tumor. Then we weigh the tumor and calculated the tumor inhibition rate by tumor inhibition rate= (average weight of control group-average weight of experimental group)/average weight of control group × 100%. All data were analyzed by one-way analysis of variance using SPSS. The difference between two groups was analyzed by the Student-Newman-Keuls test. Standard deviation was represented by error line. *P < 0.05 as compared to the saline group. ADM, doxorubicin or adriamycin; YSL, tripeptide tyroserleutide.

The groups treated with low-dose doxorubicin were observed for 60 successive days. The tumor inhibition rate of YSL alone group and YSL combination group were 37.11% and 52.17%, respectively, higher than that of the low-dose doxorubicin group (30.14%). The tumor inhibition rate of the YSL combination group was significantly different as compared to the saline group (P < 0.05), but not as compared to the doxorubicin group (P > 0.05; Figure 8).

## Discussion

Doxorubicin is a broad-spectrum antibiotic used widely for its anti-tumor activity. It is considered one of the most useful anti-tumor drugs available [6]. However, it's clinical use is limited by its serious side effects including bone marrow depression [7], liver and kidney injury [8,9], and cardiac toxicity [10]. Cardiac toxicity is considered the main side effect; some consider doxorubicin's dose-dependent cardiac toxicity to be more dangerous than its other serious side effects [11]. When doxorubicin is combined with other chemotherapeutic agents such as cyclophosphamide, bleomycin, and cisplatin, doxorubicin's cardiac toxicity is amplified. Therefore, doxorubicin dosage cannot be escalated and its use in combination therapy is limited. Identifying an acceptable therapeutic partner for doxorubicin remains an area of considerable clinical interest.

YSL is novel anti-tumor peptide that can inhibit the growth of human hepatocellular carcinoma cells without observable side effects. High performance and low toxicity are characteristic of YSL, and have become the basis efforts to use YSL and doxorubicin combination therapy to cure hepatoma. In this study, we imitated the clinical conditions of doxorubicin therapy and examined the therapeutic efficacy of YSL in combination with different dosages of doxorubicin. We evaluated the effects on the survival in a nude mouse model of human hepatocellular carcinoma.

Changes in tumor volume and weight are the most direct indices for judging the efficacy of anti-tumor therapy. We found that the tumor inhibition rate was enhanced when YSL was combined with mid- or low-dose doxorubicin, demonstrating that YSL enhances the anti-tumor effect of doxorubicin. In particular, the tumor inhibition rate of YSL plus low-dose doxorubicin was higher than that of the doxorubicin alone, whereas there was no statistically significant difference between the doxorubicin and saline groups. The manifestation of therapeutic action when low-dose doxorubicin is given in conjunction with YSL versus no therapeutic action when given alone suggests that YSL synergizes with doxorubicin. The inclusion of YSL can reduce the dosage required to obtain the clinical benefit of doxorubicin while limiting the potential side effects. These data are consistent with the principle of combined therapy for tumor treatment [12].

Other key indices for evaluating tumor therapy efficacy are survival time and survival quality. Research about survival quality has received increasing attention in the international community in recent years. Survival quality (quality-of-Life, QOL) has been widely used as an index to evaluate therapeutic results [13,14]. At the 2005 meeting of the American Society of Clinical Oncology (ASCO), more than 98 articles about survival quality were presented. The completely new indexes-elevate survival quality and prolong survival time on tumor therapy curative effect was introduced clearly at ASCO 2007. In clinical anti-tumor therapy, elevating patient survival quality includes malignant tumor rehabilitation care [15], palliative treatment [16], and attenuation and synergy of radiotherapy and chemotherapy [17].

One focus of our research is on the attenuation and synergistic effects of YSL in combination chemotherapy, and the superiority of YSL for treating hepatoma. We first observed the attenuating effects of YSL in combination with deadly dosages of doxorubicin. High dosages of doxorubicin (6 mg/kg every other day) can induce death in mice; however, adding YSL to high-dose doxorubicin significantly prolongs the survival time of nude mice bearing tumors as compared to doxorubicin alone. These data suggest that YSL enhances nude mice's tolerance to doxorubicin, thereby increasing doxorubicin's clinical value. YSL in combination with mid-dose doxorubicin improves the survival status of nude mice bearing tumors and decreases doxorubicin-associated side effects such as weight loss, decreased body temperature, and reduced activity level. These data suggest that YSL plus doxorubicin both prolongs the survival time of nude mice bearing tumor and enhances their survival quality, which has important clinical implications.

Bone marrow depression is a common side effect of doxorubicin, and the influence on leukocyte counts is the most obvious. About 60–80% of patients have leukocyte depression after 10–15 days of doxorubicin therapy, and they recover by about day 21 of therapy [18]. YSL has strong protective effect on doxorubicin-induced leukocyte depression. In this study, YSL given in combination with mid-dose doxorubicin increased the number of leukocytes; thus, the inclusion of YSL in doxorubicin treatment regimens could reduce rate of infectious complications.

During therapy, doxorubicin can induce fatal cardiac toxicity, and doxorubicin-induced liver and kidney injury can be life threatening. Mid-dose doxorubicin can injure the heart, liver, and kidneys to varying degrees. We found that the degree of injury was reduced in the YSL combination group as compared to the doxorubicin group; that is, YSL seemed to prevent or repair doxorubicin-induced heart, liver, and kidney injury. The cardio- and hepatoprotective effects of YSL were especially obvious. In the YSL combination group the structure of cardiocyte was clear, hepatic lobules was complete, and the structure of the kidney cortical labyrinth and medullary ray was clear compared to the doxorubicin group.

The elevated doxorubicin levels in the blood conduce to reaching a high concentration in tumor tissue to increase the anti-tumor effects of doxorubicin [19]. However, some organs with abundant blood supply such as heart, liver and kidney will be damaged because of high doxorubicin concentration. In this study, we found that high- and mid-dose doxorubicin had obvious anti-tumor effects on human hepatocellular carcinoma BEL-7402 cells, but doxorubicin also influenced the common status and basic vital signs of nude mice bearing tumors severely. Doxorubicin's side effects include hypo-leukocytosis, cardiac injury, liver and kidney toxicity, weight loss, and survival time decurtation. Compared with control group, low-dose doxorubicin induced less severe side effects, but had no significant inhibition on the growth of human hepatocellular carcinoma BEL-7402 cells in nude mice. Combination therapy with YSL significantly protected the mice from doxorubicin-induced side effects and increased the anti-tumor effects of doxorubicin. YSL could induce the apoptosis of human hepatocellular carcinoma BEL-7402 cells [20]. And Xu et al found that tumor necrosis factor-related apoptosis inducing ligand (TRAIL) could significantly increase the anti-tumor effects of doxorubicin through inducing the apoptosis of cancer cells [21]. So we think that the mechanisms enabling YSL to augment the cytotoxicity of doxorubicin may correlate well with the increased ability of doxorubicin when combined with YSL to induce apoptosis. Also YSL significantly increased the expression of PTEN in human hepatocellular carcinoma BEL-7402 cell. Over-expression of PTEN could increase the sensitivity to doxorubicin in human breast cancer MCF-7 cells [22]. The boosting of activity of doxorubicin when combined with YSL may result from the increased expression of PTEN to increase the sensitivity of BEL-7402 cells to doxorubicin.

In addition, our study observed the effects of co-administration of YSL and high-, mid-, and low-dose doxorubicin on nude mice bearing tumors. Although few side effects were recorded in low-dose doxorubicin group, there was no significant difference in tumor volumes and weights between the low-dose doxorubicin group and saline group. The tumor inhibition rate of YSL combination group was higher than that of the low-dose doxorubicin group, significantly different as compared to the saline group. YSL might enhance the efficacy of doxorubicin and have a dosage sparing effect which can reduce the dosage of doxorubicin to reduce its side-effects. Hence, it is possible that YSL can act as a new anti-tumor drug for the clinical development of combination with doxorubicin. Also YSL could reduce doxorubicin-induced side effects in combination with mid- and high-dose doxorubicin which produced significant anti-tumor effects. For example YSL prolonged the survival time of nude mice bearing tumor and overcame the negative side effects of doxorubicin by protecting hematological and vital organ health. Doxorubicin can undergo redox cycling to produce reactive oxygen species. The oxidant-generating activity of doxorubicin is thought to be responsible for the side effects of the drug [23]. Further research needs to be carried out to test whether YSL would interfere with oxidant-generating activity of doxorubicin to inhibit side effects of drug. Moreover, the mechanism accounting for side-effects of doxorubicin mainly depends on its cytotoxicity. Further experiment is required to show that YSL could selectively induce apoptosis of human hepatocellular carcinoma cells but not normal cells from various organs.

## Conclusion

In this study, the potential attenuation and synergistic effect of tripeptide tyroserleutide plus doxorubicin against human hepatocellular carcinoma in nude mice were investigated. Co-administration of YSL and low-, mid-, or high-dose doxorubicin respectively decreased chemotherapy-associated weight loss, leukocyte depression, and heart, liver, and kidney damage as compared to doxorubicin alone. Although there were no statistically significant differences in tumor weights between doxorubicin alone group and YSL plus doxorubicin group, YSL plus doxorubicin produced greater tumor inhibition rate than doxorubicin. The combination of YSL plus doxorubicin increased the sensitivity of tumors to chemotherapeutic drugs and reduces the side effects associated with doxorubicin chemotherapy.

## Abbreviations

YSL: tyroserleutide; ADM: Doxorubicin or Adriamycin; IP: intraperitoneally; HE: hematoxylin and eosin; SD: standard deviation; ANOVA: analysis of variance; ASCO: American Society of Clinical Oncology; TRAIL: tumor necrosis factor-related apoptosis inducing ligand; WBC: white blood cells; PLT: platelets; Hb: hemoglobin.

## Competing interests

The authors declare that they have no competing interests.

## Authors' contributions

ZZF and CLJ contributed to perform the animal study. LR designed the study, JJ contributed to the statistical analysis. LY was in charge of histopathology examinations. XQ and ZCL contributed to the data collection. WL was in charge of blood counts. WS was in charge of the tumors' collection. YZ wrote the manuscript.

All authors read and approved the manuscript.

## Pre-publication history

The pre-publication history for this paper can be accessed here:


